# Role of Demyelination in the Persistence of Neurological and Mental Impairments after COVID-19

**DOI:** 10.3390/ijms231911291

**Published:** 2022-09-25

**Authors:** Marina Y. Khodanovich, Daria A. Kamaeva, Anna V. Naumova

**Affiliations:** 1 Laboratory of Neurobiology, Research Institute of Biology and Biophysics, Tomsk State University, 634050 Tomsk, Russia; 2 Laboratory of Molecular Genetics and Biochemistry, Mental Health Research Institute, Tomsk National Research Medical Center of the Russian Academy of Sciences, 634014 Tomsk, Russia; 3 Department of Radiology, University of Washington, Seattle, WA 98109, USA

**Keywords:** COVID-19, post-COVID syndrome, long COVID, SARS-CoV-2, demyelination, MRI, neuroimaging, neurological consequences, cognitive impairment, mental sequelae

## Abstract

Long-term neurological and mental complications of COVID-19, the so-called post-COVID syndrome or long COVID, affect the quality of life. The most persistent manifestations of long COVID include fatigue, anosmia/hyposmia, insomnia, depression/anxiety, and memory/attention deficits. The physiological basis of neurological and psychiatric disorders is still poorly understood. This review summarizes the current knowledge of neurological sequelae in post-COVID patients and discusses brain demyelination as a possible mechanism of these complications with a focus on neuroimaging findings. Numerous reviews, experimental and theoretical studies consider brain demyelination as one of the mechanisms of the central neural system impairment. Several factors might cause demyelination, such as inflammation, direct effect of the virus on oligodendrocytes, and cerebrovascular disorders, inducing myelin damage. There is a contradiction between the solid fundamental basis underlying demyelination as the mechanism of the neurological injuries and relatively little published clinical evidence related to demyelination in COVID-19 patients. The reason for this probably lies in the fact that most clinical studies used conventional MRI techniques, which can detect only large, clearly visible demyelinating lesions. A very limited number of studies use specific methods for myelin quantification detected changes in the white matter tracts 3 and 10 months after the acute phase of COVID-19. Future research applying quantitative MRI assessment of myelin in combination with neurological and psychological studies will help in understanding the mechanisms of post-COVID complications associated with demyelination.

## 1. Introduction

Over the past two and a half years, the COVID-19 pandemic has completely changed people’s lives around the world. Although the infectious disease COVID-19 is primarily associated with pneumonia, numerous observations show that its causative agent, the SARS-CoV-2 coronavirus, can infect other vital organs such as the heart, kidneys and brain [1,2,3,4,5,6,7,8,9,10,11,12,13,14,15]. For the first time, the neurological symptoms of COVID-19 became known in the first months of the epidemic in Wuhan [1]. In patients with COVID-19, the CNS impairments are dizziness, headache, impaired consciousness, acute cerebrovascular disease, ataxia, and convulsions; the peripheral nervous system impairments are disturbances in taste and smell, visual impairment, and nervous pain [1]. 

As variants of SARS-CoV-2, such as omicron, have become widespread, the risk of a severe outcome has decreased [16,17] and more attention has been paid to the long-term consequences of this infection. A fairly short time after the start of the pandemic, it became clear that COVID-19 is not only a serious disease, often accompanied by a fatal outcome, but also has long-lasting systemic disorders-the so-called post-COVID, or post-COVID syndrome, or long COVID. The World Health Organization’s definition of post COVID-19 condition refers to cases of impaired health after COVID-19, which are observed for at least two months after the illness, last, and cannot be explained by other reasons [18,19]. Now long-COVID is considered as a multi-organ disorder with a wide spectrum of clinical manifestations that may be indicative of underlying pulmonary, cardiovascular, endocrine, hematologic, renal, gastrointestinal, dermatologic, immunological, psychiatric, or neurological disease [20].

Disorders of the central nervous system (CNS) are among the most severe complications that persist for many months after this infectious disease and significantly affect the quality of life. To date, neurological sequelae of varying severity or mental health changes are known to be recorded, according to various sources, in 30–80% of patients with COVID-19 [7,20,21,22,23,24,25,26,27,28], including non-hospitalized patients with mild disease [21,27]. There is accumulating clinical evidence of the COVID-19 as a risk factor of demyelination both in the peripheral and central nervous systems responsible for manifestation of neurological complications [2,13,29,30,31,32,33,34,35,36,37]. Demyelinated axons are at risk for degeneration resulting in permanent disability of the patients. Therefore, non-invasive evaluation of myelination is important for assessment of clinical complications, treatment planning and prediction of outcome. However, no studies have been conducted so far that would quantitatively assess brain myelin in post-COVID patients. Demyelination might be caused by inflammatory processes, impaired cerebrovascular flow, damage to oligodendrocytes and myelinated axons. This review summarizes the current knowledge of demyelination in post-COVID patients with a focus on neuroimaging findings and discusses possible mechanisms for demyelination occurrence.

## 2. Neurological and Mental Consequences of COVID-19

During two years following the onset of the pandemic, the neurological and mental consequences of COVID-19 were well documented in recovered patients [7,20,21,22,23,24,25,26,27,28,38,39,40,41]. Among the most observed neurological persistent symptoms are “brain fog”, fatigue, headache, myalgia, sleep disorders, impaired sense of smell and taste [7,20,21,22,23,24,25,26,27,28]. Less commonly reported neurological manifestations may include dizziness, numbness/tingling, blurred vision, movement disorders, tinnitus, seizures, dysphagia, and others [21,38,39]. In addition to obvious neurological symptoms, post-COVID is often characterized by less obvious but long-lasting impairments in the cognitive and emotional functions [21,26,27,40,41], which patients rarely seek help with. It is known that these disorders can persist for up to several months [21,23,30,39] and even a year [38] in a significant number of patients, including young and after COVID-19 of mild to moderate severity [27]. 

Table 1 summarizes the results of several studies on the incidence of neurological symptoms and mental impairment in post-COVID patients. There are differences in the percentage of occurrence of neurological consequences reported in different studies. At similar follow-up periods of 6 months [21,39,42] the percentage of occurrence differs substantially for headache (2%, 68% or 9.7%), myalgia (2%, 55% or 29.6%), and dysgeusia/hypogeusia (7%, 55% or 29.6%). Moreover, a higher percentage of impairments are reported for patients with a mild COVID-19. Differences can be explained by the peculiarities of patient selection and testing; therefore, such quantitative estimates should be treated with prudence. The same studies show the highest percentage of patients with fatigue, anosmia/hyposmia, insomnia, depression/anxiety, and memory/attention deficit. Unfortunately, there are few studies that combine the analysis of neurological symptoms with objective psychological testing of cognitive and mental functions in post-COVID patients.

Several systematic reviews and meta-analyses summarize the quantitative assessments of the neurological manifestations of post-COVID across different studies and generally support the findings of the above studies [43,44,45,46]. The work by Ceban et al. [44] included 68 studies for a meta-analysis of fatigue occurrence and 43 studies for a meta-analysis of cognitive impairment. A meta-analysis found that the percentage of individuals experiencing fatigue 12 or more weeks after being diagnosed with COVID-19 was 32%, and the percentage of individuals with cognitive impairment was 22%. Iqbal et al. [46], after analyzing 38 studies, concluded that fatigue (48%) and sleep disturbance (44%) are the most prevalent symptoms in chronic post-COVID syndrome. The most representative meta-analysis of Lopez-Leon et al. [43] summarized data of 47,910 patients from 81 studies and showed that 80% of long-COVID patients developed one or more prolonged neurological symptoms including fatigue (58%), headache (44%), impaired attention (27%), ageusia (23%), anosmia (21%), memory deficit (16%), hearing loss or tinnitus (15%), chills (7%), dizziness (3%) and stroke (3%). Anxiety and depression were observed in 13% and 12% of patients, respectively. Han et al. [45] analyzed one-year post-COVID symptoms of 8591 patients from 18 peer-reviewed studies and concluded that fatigue/weakness (28%), myalgia (26%), depression (23%), anxiety (22%), memory loss (19%), concentration difficulties (18%), and insomnia (12%) were the most frequent neuropathological changes.

## 3. Predictors of Post-COVID Neurological Complications

Most studies indicate female sex and older age as risk factors for long-term neurological consequences of the COVID-19 [39,42,47,48,49,50]. 

Elderly patients are among the most vulnerable populations facing COVID-19 infection. Cognitive complaints are frequent in elderly patients after COVID-19 [51,52] and verse versa, patients suffering from dementia are at higher risk of COVID-19 infection and higher risk of severe forms and hospitalization [53]. The meta-analysis on the risks associated with COVID-19 infection showed that suffering from dementia increases by 2 to 3 the risk of contracting COVID-19 compared with patients of comparable age [54]. Several studies showed that ApoE4 genotype, a genetic factor linked to Alzheimer’s disease, has been reported as a risk factor for more severe COVID-19 infection [55]. 

Data on the relationship of the disease severity with the likelihood of long-term consequences of COVID-19 are contradictory. A number of studies showed the association of the severity of the acute COVID-19 with the occurrence of long-term neurological consequences [23,39,42,46,48,49,50]. It was also found that the number of initial symptoms better predicts the presence of long COVID than the severity of the acute phase of the disease [47,50,56]. Other studies showed that severity of the acute phase of the disease is less important as predictor of the long-term health complications than cumulative number of the specific COVID-19 symptoms, so-called number of initial symptoms, during the first week of acute phase [47,50,56]. Miskowiak et al. [23] studied the relationship between acute phase severity and cognitive changes in post-COVID patients. The authors supposed that cognitive sequelae of COVID-19 might be associated with the severity of the lung affection and potentially restricted cerebral delivery of oxygen. 

**Figure 1:**
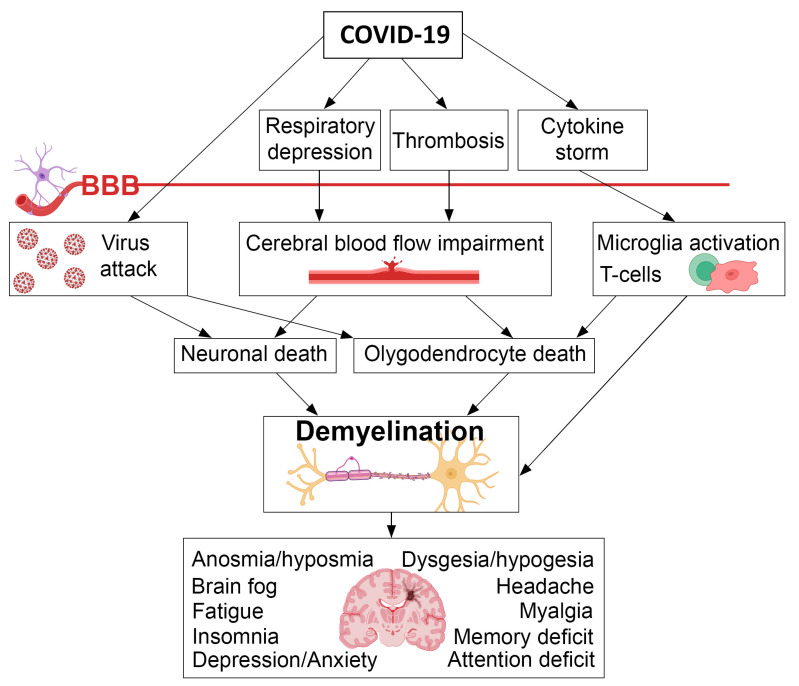
The schematic representation of the relationships between COVID-19, demyelination, neurological and mental disorders.

Studies of Pilotto et al. [42], found that not only older age and severity of COVID-19, but also comorbidities are independent predictors of long-term neurological manifestations. Sudre et al. [47] indicated importance of the body mass index as a predictor of post-COVID complications. Hypertension [50,57] and respiratory symptoms at the onset [49] are also listed as a risk factor for development of the post-COVID neurological symptoms. The relationships between COVID-19, demyelination, neurological, and neuropsychological disorders are schematically shown in Figure 1.

The physiological basis of neurological and mental disorders is still poorly understood. We did not find systematic studies in the available literature that combine the assessment of cognitive functions, the emotional sphere with the study of structural brain changes using MRI. According to a number of studies [10,29,30,57], including animal models [30], brain demyelination might be the physiological basis for these impairments. The recent review manuscript of Orier et al. [58] also suggests that COVID-19 is likely to trigger demyelination and brain cell degeneration. The following section considers demyelination as a basis of neuropsychological disorders.

## 4. Clinical Significance of Demyelination

### 4.1. Demyelinating Diseases and Mental Disorders

Neurodegenerative diseases, such as Alzheimer’s disease, Parkinson’s disease, amyotrophic lateral sclerosis, and multiple sclerosis are characterized by demyelination and inflammation caused focal and diffuse damage in both the white matter (WM) and gray matter (GM) [59,60]. Many publications have shown the direct connection between demyelinating diseases and mental disorders. Besides physical disability, cognitive deficits are present in 40–70% of patients with multiple sclerosis affecting visual and/or verbal efficiency [61,62]. Combined MRI/PET study showed that lower myelin content by 11C-PIB uptake was associated with worse cognitive status in multiple sclerosis [63]. MRI studies have shown that demyelination was greater in subjects with Alzheimer’s disease and vascular dementia in comparison compared with similar age control group and myelination deficits correlated with cognitive decline and impairment [64]. 

Predominantly structural brain characteristics extracted from brain imaging techniques such as total brain volume, cortical GM volume as well as WM lesion volume were found to be related to cognitive performance and could predict cognitive decline [65,66]. Especially GM atrophy correlating with cognitive impairment [67,68,69]. 

### 4.2. Demyelination and Aging

For a long time the loss of cortical neurons was considered as the main cause of human cognitive decline during normal aging, since numerous volumetric studies showed both gray and white matter atrophy with age [70,71,72]. One of the most representative volumetric studies by DeCarli et al. [73] performed on 2200 individuals aged 34 to 97 showed that age-related differences are generally small prior to age 50 but increase thereafter in the frontal and temporal lobe (approximately 12% and 9% decline with age respectively). 

Detailed histopathological studies of elderly people without Alzheimer’s disease show that the number of cortical neurons was not significant in most regions of the aging neocortex [74,75] and hippocampus [76]. Several studies reviewed in [77] also have shown a relatively greater age-associated decline in white matter volume or the presence of white matter volume loss in the absence of grey matter loss. These results directed efforts to find other causes of cognitive decline with age, in particular changes in nerve fibers.

Histopathological studies of elderly individuals showed that myelin sheaths undergo degenerative changes, such as the formation of splits containing electron dense cytoplasm, and the formation on myelin [78,79,80,81]. Observations of white matter degradation also include myelin pallor, loss of myelinated fibers, and malformation of myelin sheaths [77,82].

It was suggested that such degenerative changes lead to cognitive decline because they cause changes in conduction velocity, resulting in a disruption of the normal timing in neuronal circuits [78]. These assumptions have been confirmed in several studies. O’Sullivan et al. [83] have used DTI to estimate age-related alterations in white matter and found linearly reduced diffusional anisotropy, which is a marker of white matter tract integrity, in old subjects. The white matter disruption was maximal in frontal regions and the authors consider the cortical disconnection as the basis for the age-related cognitive decline in normal aging. Quantitative myelin imaging study had shown greater myelin content in young participants in comparison to the old unimpaired participants [64]. This is in good agreement with changes recently observed in healthy aging [84,85].

By using large datasets of brain images of healthy individuals, a machine learning model can be trained to estimate the age of a given brain [86]. The model can provide the best guess of the age of that person’s brain, that is, the ‘brain age’, which can look older or younger than the actual, chronological age of that person. In several brain disorders [87], including MS [87,88,89], brains typically look older than those of their healthy peers.

Despite the fact that myelination in humans is mostly completed by the age of 10 [90,91], myelin renewal continues for a long time into adulthood and is important for the repair of damage to the myelin sheaths, neural circuit plasticity, and cognitive learning [80]. The remyelination process depends on the ability of oligodendrocyte progenitor cells (OPCs) to proliferate, migrate, and finally differentiate into myelinating oligodendrocytes.

Recent studies have shown that, during the aging process, there is a gradual de-crease in the regenerative capacity of OPCs. Before differentiating into mature myelinating oligodendrocytes, NG2-positive OPCs exhibit differentiation phase characterized by the expression of the G-protein-coupled receptor subtype GPR17 [92]. OPCs can be divided into two functionally distinct pools: slowly dividing NG2-positive OPCs, which have a self-renewal property similar to stem cells [93], and GPR17-positive COPs, which differentiate into myelinating oligodendrocytes when necessary [94,95]. Both pools were found to decrease significantly in the aging brain [96]. Dysregulation of OPCs and COPs leads to impaired replacement of myelin lost during aging [96,97], which is a key factor in age-related decline in neural network plasticity and cognitive function [98], as well as myelin loss in AD [85] and remyelination failure in chronic MS [99,100]. The studies by Rivera [79,80,85] showed that oligodendroglial genes are among the most dysregulated during the aging and this dysregulation is critically important for the successful transition of OPCs into myelinating oligodendrocytes.

## 5. Possible Mechanisms of Demyelination Caused by COVID-19

Demyelination, which is the destruction of the myelin sheath of the axon, can occur for many reasons. Primary demyelination, when the cause of the disease is precisely the destruction of myelin, is classified into several categories according to pathogenesis [101]: (1) demyelination caused by inflammatory processes, (2) viral demyelination, (3) demyelination caused by acquired metabolic disorders, (4) hypoxic-ischemic forms demyelination, (5) demyelination due to focal compression. Some of these reasons are related to COVID-19 and will be discussed below.

### 5.1. Inflammation and Autoimmune Response

Demyelination caused by inflammation is usually the result of an attack by the own immune system on the myelin sheath [102,103]. In primary demyelinating diseases, the causes of the disease remain unknown. However, most researchers consider the autoimmune mechanism to be the main one: autoreactive T-lymphocytes penetrate the blood-brain barrier from the peripheral circulation and trigger an inflammatory cascade leading to demyelination [104].

It is assumed that this process can be initiated by viral and bacterial factors penetrating the nervous tissue. Viral or bacterial proteins on oligodendrocyte membranes and myelin sheaths cause primary T-cell activation [102,104,105]. In turn, T-cells release cytokines that cause B-cells to form autoantibodies and attack the myelin sheath and oligodendrocytes [106]. In addition, cytokines trigger the activation of microglia, macrophages, and astrocytes. As a result, microglial and astroglial cells also begin to secrete inflammatory cytokines. In addition, microglia often play the role of antigen-presenting cells that enhance the development of a pathological immune response in MS. As a result of this autoimmune response and local vascular inflammation, myelin sheaths and oligodendrocytes are injured [60,104,105]. 

Thus, increased production of pro-inflammatory cytokines by T-cells, microglia and macrophages is a key event in the development of an autoimmune response leading to demyelination. 

A similar picture is observed in COVID-19 patients. It is widely known that the acute phase of COVID-19 is accompanied by the phenomenon of the so-called cytokine storm, characterized by high levels of pro-inflammatory cytokines in the blood serum [107], which leads to an overactive immune response in host tissues [108,109]. Nearly all elements of the inflammatory cascade observed in MS have been found in COVID-19 patients. It was revealed that cytotoxic T lymphocytes and pro-inflammatory cytokines can cross the blood-brain barrier, affecting CNS innate immune cells such as macrophages, microglia, and astrocytes, and inducing their pro-inflammatory state, stimulate microglial activation and trigger immune-mediated demyelination [36,110,111]. Many of these cytokines such as interleukin 6, IL-2 T cell growth factor, tumor necrosis factor alpha (TNF-α) are also involved in the regulation of signaling pathways in the development of multiple sclerosis [112,113]. Finally, post-mortem histopathological studies have also demonstrated myelin loss in COVID-19 patients [111].

Further evidence of similarities with demyelinating diseases is the formation of autoantibodies, an essential component of the autoimmune response, in post-COVID patients [114,115,116,117,118,119,120,121], which may indicate a loss of immunological tolerance [115]. I was found that the titer of autoantibodies positively correlates with the severity of the disease [114]. Although there are few studies on the composition of autoantibodies in COVID-19, some of them revealed the formation of antibodies that attack components of the myelin sheath. Johnsson M et al., report a series of SARS-CoV-2 positive patients that developed a rare disease, myelin oligodendrocyte glycoprotein-associated disorder, which was characterized by the presence of antibodies to myelin oligodendrocyte glycoprotein [122]. In addition, patients with COVID-19 have high serum levels of antiphospholipid antibodies [120,121], which can target the lipidic components of myelin [123,124,125].

It is important to note that a high level of cytokines and an autoimmune response is observed not only in the acute phase of COVID-19. A recent study showed that the autoimmune response to COVID-19 persists for 3–5 months after the infection and therefore may cause long-term consequences of the disease [8]. The long-term persistence of high titers of pro-inflammatory cytokines, chemokines, growth factors and endothelial activation for 3–6 months after the disease in post-COVID patients has been described [126].

A recent study by Fernandez-Castaneda et al. [30], performed on a mild respiratory COVID model in mice and histopathological human samples, cogently demonstrated the inflammatory mechanism of post-COVID demyelination and its possible influence on cognitive impairment. The authors found white-matter-selective microglial reactivity in mice and humans after respiratory SARS-CoV-2 infection. Additionally, in a mouse model, the authors revealed persistently impaired hippocampal neurogenesis, loss of oligodendrocytes and myelin along with elevated level of cytokines/chemokines. 

Finally, attention should be paid to the similarity of symptoms and predictors of long COVID and demyelinating diseases. Both demyelinating diseases and long COVID are characterized by fatigue, delirium, depression, cognitive and sensory impairment [7,67,127]. For both diseases, female sex is a risk factor [39,42,47,48,49,50,128]. However, it should be noted that there is some discrepancy between the predictors of MS and the cytokine storm, which can be considered as a mechanism of post-COVID demyelination. MS is usually diagnosed in young adults (average age of onset is 30 years [60]) and more often in women [60,128], while the predictors of a cytokine storm are male sex and age over 40 years [129].

Among the comorbidities that increase the severity of COVID-19 are many diseases that are closely related to disorders of the immune system. Patients with autoimmune diseases are known to be more vulnerable to severe outcomes from COVID-19 [130]. In addition, modulation of the immune response has been shown to be one of the mechanisms responsible for the severe acute phase and increased mortality in comorbidities such as cardiovascular disease, cancer, diabetes mellitus, nonalcoholic fatty liver disease, chronic hepatitis B, and chronic kidney disease [130,131].

### 5.2. Direct Effect of the Virus on Oligodendrocytes

Another cause of demyelination might be related to the direct effect of the virus on oligodendrocytes, leading to disruption of their function and cell death [101,132]. For example, progressive multifocal leukoencephalopathy is a progressive demyelinating disease caused by human polyomavirus 2 (JC virus) [133]. Virus reactivation occurs under conditions of weakened immunity [134] and affects motor functions, speech, vision, and cognitive abilities [101,133]. In the active demyelinating lesions, infected oligodendrocytes and astrocytes with altered morphology are found [133]. In contrast to astroglia and neurons [15], the effect of SARS-CoV-2 virus on the functionality and survival of oligodendrocytes, has not yet been studied; however, a number of facts indicate a high probability of such an effect. First, it has been shown that SARS-CoV-2 crosses the blood-brain barrier (BBB) [135]. Second, a recent experimental study showed that, although oligodendrocytes survive after infection with the neurotropic coronavirus, they begin to express genes that contribute to chronic inflammation, while being located near demyelinated areas [136]. It has also been shown that a number of coronaviruses (229-E, HCV-OC43) are capable of infecting oligodendrocytes in cell culture, suppressing the production of PLP, and causing their apoptosis [12]. 

### 5.3. Cerebral Blood Flow Impairment

Impairment of the cerebral blood flow is the cause of the many pathological events in the brain, including loss of neurons, edema, axonal death, demyelination, neuroinflammation, and glial scarring. It is known that oligodendrocytes with their intensive metabolism are extremely sensitive to the lack of oxygen and glucose [137,138] and therefore are often responsible for the severity of ischemic-hypoxic damage [101]. Myelin destruction is following the death of neurons and axons [139]. Based on histological studies, the destruction of myelin can be observed as early as 30 min after the ischemic episode [137,140,141]. Our recent animal studies using MRI and histological methods have shown that demyelination after stroke modeling is observed from the first day [139] and persists up to three months after surgery [142]. Clinical studies of ischemic stroke patients show that chronic structural damage of the white matter is a risk of poor outcome [143]. In patients with severe cerebrovascular disease of small vessels, subcortical white matter is often demyelinated and is associated with dementia and neurological disorders [101]. Brain demyelination is observed in patients due to global hypoxia after cardiac arrest, asphyxia, or depression of cardiorespiratory function [144]. In an animal study, we also showed the demyelination of the hippocampus in rats after total cerebral ischemia, which simulates cardiac arrest [145].

COVID-19 related decrease in lungs functionality and respiratory depression lead to hypoxia, thromboembolism with microbleeds and brain ischemia [3,7,10,146]. According to the study of Varatharaj et al. [22], more than 60% of the hospitalized patients with COVID-19 have cerebrovascular complications. It has also been shown that COVID-19 is an independent risk factor for acute ischemic stroke [147]. As noted above, demyelination always accompanies the death of neurons and axons in ischemic-hypoxic damage, but it can also be a separate factor due to the death of oligodendrocytes.

Therefore, a number of studies indicate that SARS-CoV-2 virus induces myelin damage, oligodendrocytes death and disruption of neurological function as result of impaired respiration leaded to hypoxia, cerebral ischemia and inflammatory response to viral infection.

## 6. Demyelination in COVID and Post-COVID Patients

Brain demyelination after COVID-19 has been discussed in several reviews as potential mechanism of the neurological complications [10,13,29,35,36,148]. Among the complications of coronavirus infection diagnosed by MRI and associated with demyelination, encephalomyelitis, meningoencephalitis, strokes, Guillain-Barré syndrome, polyneuropathy, encephalopathy, and multiple sclerosis are noted [2,11,31,32,33,34,37,146,149,150,151,152,153]. However, there is a lack of clinical MRI studies that allow us to estimate the incidence of demyelination in patients with acute or long COVID. Most of these studies, with rare exceptions, are case reports [2,32,33,34,37,146,149,150] or a description of a number of cases [151,152,154,155,156,157,158]. We found only two systematic MRI studies of patients with mild to moderate disease, in which demyelination was assessed quantitatively [5,57]. Here, we will focus on neuroimaging of reported cases of demyelination due to COVID-19 and attempt to estimate the rate of detection of demyelination by conventional MRI. Table 2 shows original MRI studies (excluding case reports) that quantified demyelination or at least provided information on the number of patients in whom it was found.

Most of the studies reporting demyelination, found mainly by FLAIR hyperintensity, are performed in patients in the acute phase of COVID-19 (Table 2). In these studies, imaging protocol usually included T1-weighted (T1w) imaging, fluid-attenuated inversion recovery (FLAIR) imaging, susceptibility-weighted imaging (SWI), perfusion imaging (PWI), diffusion-weighted imaging (DWI) with apparent diffusion coefficient (ADC) map construction, and contrast-enhanced spin-echo T1-weighted (Gd-T1w) imaging.

The most frequent MRI findings included FLAIR of T2 hyperintensities located in cortical, deep, and subcortical white matter (WM), corpus callosum, or periventricular regions. In a retrospective study, Chougar et al. [151] analyzed data from hospitalized patients with severe COVID-19 who underwent an MRI scan; only in 4 of 73 patients were multiple demyelinating lesions detected by FLAIR hyperintensities found in the periventricular cortical white matter (WM), in the corpus callosum and basal ganglia. One patient had corticospinal tracts FLAIR hyperintensity. Unfortunately, these changes were not evaluated quantitatively. According to inclusion criteria, all 73 patients had de novo (no previous history of neurologic disease) neurologic symptoms.

Another retrospective study by Mahammedi et al. [152] examined 108 hospitalized patients with acute phase of severe COVID-19; 20 of them underwent conventional MRI with and without contrast. In 7 patients “nonspecific but likely chronic” FLAIR hypointensities were found, which potentially point to demyelination. Besides, in one patient multiple sclerosis (MS) plaque exacerbation was detected, 2 patients showed basal ganglia and 3 patients showed subcortical WM disease. The most common neurologic symptoms were altered mental status in 64 of the 108 patients (59%) and ischemic stroke in 34 (31%).

Kremer et al. [156] investigated 37 severe patients with neurological manifestations and abnormal MRI in the acute phase of COVID-19. The most frequent MRI findings were: signal abnormalities located in the medial temporal lobe in 16 patients, non-confluent multifocal FLAIR WM hyperintense lesions, with variable enhancement, with associated hemorrhagic lesions in 11 patients, and extensive and isolated WM microhemorrhages in 9 patients.

Radmanesh et al. [157] found abnormal WM T2 hyperintensities in 10 of 27 critically ill patients with COVID-19 underwent brain MRI. Four patients had only diffuse leukoencephalopathy, one patient had only microhemorrhages, and 6 patients had a combination of both. T2 hyperintensities were symmetric and confluent, demonstrated mild restricted diffusion, and involved bilateral deep and subcortical WM. During the 3–5 weeks after brain MRI, six of the 11 patients died (three had leukoencephalopathy, one had microhemorrhages, and two had both).

Scullen et al. [158] investigated critical patients with COVID-19 with de novo neurologic manifestations. 27 of 76 patients (35.5%) had evidence of new-onset neurologic disease, which ranged from mild headache and dysgeusia to severe focal deficit; 26 patients had altered mental status. MRI findings of 6 from 27 explored patients included FLAIR hyperintensities of different location: deep white matter, the corpus callosum, basal ganglia, bilateral mesial temporal structures, lenticular nuclei, cerebral peduncle, and centrum semiovale.

Several studies report demyelination in the cortex in patients with acute COVID-19. Anzalone et al. [154] reported 4 cases of subacute encephalopathy occurring in 21 patients with SARS-CoV-2 infection presenting with neurological symptoms studied with brain MRI. In these patients, a multifocal involvement of the cortex appeared hyperintense on T2-weighted and FLAIR images and were located in the parietal, occipital and frontal regions. No significant imaging changes were found in other MR images. Nicholson et al. [155] also observed multifocal subcortical/cortical petechial-type hemorrhages, SWI extensive multifocal abnormalities, and FLAIR hyperintensity along some cortical regions in severe 4 patients in the acute phase of COVID-19.

In retrospective study, Klironomos et al. [159] observed 43 hospitalized patients with severe COVID-19 using a detailed standardized protocol for brain MRI (Table 2). WM changes were observed in 23 of 41 patients, the most common were confluent, symmetric, and periventricular lesions, juxtacortical WM lesions, changes in the middle cerebellar peduncles and corpus callosum. Interestingly, T2w FLAIR signal enhancement in the olfactory bulbs and tracts was observed in 7 of 37 patients; in two patients, there was also subtle contrast enhancement. 

We found only two studies assessed demyelination quantitatively using DTI and fractional anisotropy parameter (FA). Qin et al. [5] performed MRI study in patients with mild and moderate course of the disease without severe neurological symptoms in the acute stage. Along with a decrease in the thickness of the cortex and the volume of a number of subcortical formations, changes in the microstructure of the cerebral cortex were detected using diffusion tensor imaging (DTI) and the fractional anisotropy parameter (FA). The cognitive and neurological impairment of the patients was not studied. 

Tian et al. [57] explored the cross-sectional and longitudinal consequences of COVID-19 from 3 to 10 months after the acute phase in 34 patients without neurological manifestations. MRI protocol included conventional MRI, structural 3D T1w images, 3D pseudocontinuous arterial spin labeling (3D-pcASL), and high-resolution DTI. In addition to positive dynamic of cortical thickness and hypoperfusion in severe cases, the authors found that subcortical nuclei and white matter differences between groups and within subjects showed various trends, including recoverable and long-term unrecovered differences. After a 10-month recovery period, a reduced volume of nuclei in severe cases was still more extensive and profound than that in mild cases. 

The study of Marcic et al. [160] conducted in 40–60 days after recovery from mild (*n* = 16) or moderate (*n* = 23) SARS-CoV-2 infection showed multiple punctate hyperintensities on brain T2 and FLAIR MRI. Those changes were not present on pre-COVID MRI brain scans of the same patients and correlated with the severity of the clinical symptoms. The study emphasized the neurotropism of the virus and necessity to conduct the longitudinal assessment of COVID-19 consequences.

**Table 2:** Demyelination-related MRI findings in COVID and post-COVID patients.

MRI Technology	Time from Onset	Neurologic Symptoms/Diagnosis	Demyelination/ Sample Size	Demyelination-Related MRI Findings	Reference
T2-FLAIR, T1w, DTI (FA)	3 months after COVID-19	No specific neurological manifestations at the acute stage	19 mild, 32 severe/82	No obvious lesions on the conventional MRI, decreases in volume, length, and the mean FA in subcortical WM tracts in severe compared to mild patients, and in mild patients compared to controls	Qin et al. [5]
Conventional MRI, 3D T1w, 3D-pcASL, DTI (FA)	3–10 months after COVID-19	No specific neurological manifestations at the acute stage	13 mild, 21 severe/34	The trends in volume of subcortical nuclei and white matter tracts were different for 3–10 months period in patients with mild and severe COVID-19	Tian et al. [57]
T1w, Gd-T1w FLAIR, DWI, ADC	Acute COVID-19	Late awakening after withdrawal of sedation, acute neurologic symptoms	5/73 (7%)	Multiple bilateral WM deep and periventricular, corpus callosum and basal ganglia lesions in patients with severe COVID-19	Chougar et al. [151]
T1w, Gd-T1w T2/FLAIR	Acute COVID-19	Acute neurologic symptoms during hospital stay	1/20 (5%)	MS plaque exacerbation	Mahammedi et al. [152]
			7/20 (35%)	Nonspecific T2/FLAIR hyperintensity	
			3/20 (15%)	Subcortical white matter lesions	
T2w, DWI, FLAIR, SWI	Acute COVID-19	Agitation, spatial disorientation, seizure/Subacute encephalopathy	4/21 (19%)	Multifocal laminar cortical brain lesions detected by FLAIR hyperintensity	Anzalone et al. [154]
DWI, SWI, T2w, FLAIR,	Acute COVID-19	Abnormal mental status/ Thromboembolism, microbleeds, arterial microvascular thrombosis	4/*	Mild FLAIR hyperintensity along some cortical regions	Nicholson et al. [155]
T1W, Gd-T1w, DWI, gradient-echo T2, SWI, FLAIR	Acute COVID-19	Alteration of consciousness, pathological wakefulness, confusion, agitation	11/37 (30%)	Non-confluent multifocal WM hyperintense lesions on FLAIR with variable enhancement	Kremer et al. [156]
T2W, DWI, FLAIR	Acute COVID-19	Diminished mental status/Diffuse leukoencephalopathy	10/27 (37%)	Abnormal T2 hyperintensities bilateral deep and subcortical WM	Radmanesh et al. [157]
FLAIR, SWI, DWI, MRA	Acute COVID-19	De novo acute neurologic symptoms/Encephalopathy (74%), acute necrotizing encephalopathy (7%), and vasculopathy (19%).	6/27 (22%)	FLAIR hyperintensities in deep WM, the corpus callosum, and the basal ganglia	Scullen et al. [158]
SWI, DWI, Gd- DSC-PWI, ASL-PWI, T2w, Gd-T2w, FLAIR, Gd- FLAIR, T1w/TSE, Gd-T1w/TSE, T1w/GRE IR, Gd-T1w/GRE IR MRA	Acute COVID-19 and follow up	Acute neurologic symptoms during hospital stay/leukoencephalopathy, encephalopathy, hypoxic/metabolic changes, encephalitis	23/41 (53%)	Confluent, symmetric, periventricular juxtacortical WM lesions, changes in cerebellar peduncles, corpus callosum, olfactory bulbs and tracts	Klironomos et al. [159]
FLAIR, SWI, DWI, MRA	Long COVID-19	Smell and taste dysfunction, vertigo, headache, dizziness, fatigue	16 mild, 23 moderate/39 (100%)	Hyperintense lesions on FLAIR, microhemorrhage on SWI	Marcic et al. [160]

* A total number of examined patients have not been specified. Note. ASL-PWI-arterial spin labeling, DWI-diffusion-weighted imaging, PWI-perfusion-weighted imaging, DSC-PWI-Dynamic susceptibility contrast perfusion, DTI-diffusion-tensor imaging, FA-fractional anisotropy, FLAIR-fluid-attenuated inversion recovery, Gd-gadolinium-based contrast agent, GRE-gradient-recalled echo, IR-inversion recovery, SWI-susceptibility-weighted imaging, 3D-three-dimensional, GRE-gradient-recalled echo, TSE-turbo spin echo, IR-inversion recovery, MRA-magnetic resonance angiography, WM -white matter.

The majority of the imaging publications on COVID-19 are case reports or case series that demonstrate severe neurological symptoms on hospitalized patients (usually in the intensive care units). In those studies, patients with previous history of neurological disease fit into the exclusion criteria and were not imaged. Therefore, all imaging findings related to demyelination and presence of neurological symptoms are de novo consequences of the COVID-19.

Demyelination was found mainly as multifocal FLAIR hyperintensity in the cortex, corpus callosum, subcortical nuclei, cortical and subcortical WM, deep grey matter, and periventricular regions. The incidence of such lesions varies from 7 to 37%, even among severe patients with neurological symptoms. Only two systematic studies using quantitative MRI (DTI and FA) [5,57] demonstrated demyelination in a range of white matter structures in severe and mild patients 3–10 months after COVID-19 without neurological manifestations in the acute phase.

## 7. Discussion

The long-term neurological and mental (including cognitive, emotional impairments and insomnia) consequences of COVID-19 have been confirmed by numerous studies, and at present their prevalence and impact on a person’s quality of life is beyond doubt. The most persistent and common neurological and mental manifestations of long COVID include fatigue, anosmia/hyposmia, insomnia, depression/anxiety, and memory/attention deficit. The physiological basis of neurological and psychiatric disorders is still poorly understood. Many studies report only neurological sequelae, or cognitive or psychiatric impairments, or MRI abnormalities in acute or long COVID-19 patients. A very limited number of studies included all these elements together in search of the correlations and predictors.

Numerous reviews and theoretical articles consider brain demyelination as one of the likely mechanisms of neurological complications [10,13,29,35,36,148]. Probable mechanisms of demyelination are inflammation and autoimmune response, direct effect of the virus on oligodendrocytes, disrupting their function, and cerebrovascular disorders, inducing myelin damage and oligodendrocyte death. There is a contradiction between solid fundamental basis underlying mechanisms of demyelination caused by COVID-19 and relatively little published clinical evidence. Probably, the reason for this lies in the fact that most clinical studies used conventional MRI techniques, which are mostly qualitative and can detect only large, clearly visible demyelinating lesions. Standard MRI techniques cannot detect eye-invisible changes in gray matter myelination, as well as diffused changes in global myelination without focal localization. Such subtle changes can only be detected using high-precision quantitative MRI methods specific for myelin. Only two published studies used quantitative MRI techniques (DTI and FA) to study demyelination after COVID-19 [5,57]. Dynamic changes in white matter tracts were detected in patients recovered after severe and mild infection without neurological symptoms in the acute phase; changes in myelination between mild, severe COVID and healthy control were significant [5,57]. Unfortunately, these studies did not examine cognition and function using objective psychological tests. Therefore, it is difficult to make conclusions regarding the involvement of demyelination in neurological complications of the COVID-19.

Diffusion tensor imaging (DTI) and its parameters, fractional anisotropy (FA) and radial diffusion (RD), are among the long-used methods for quantifying myelin [161]. This method is well suited for assessing and reconstructing white matter tracts but is not without its drawbacks in terms of myelin quantification. The problem with using DTI to assess myelin is the sensitivity of FA to the direction of diffusion in areas containing multidirectional fibers. There are several non-conventional, actively developed methods of myelin quantification, such as myelin water fraction (MWF) [162,163,164], based on multicomponent analysis of T2 relaxation curves, as well as inhomogeneous magnetization transfer (ihMT) [165,166,167] and molecular proton fraction (MPF) mapping [168,169,170,171,172,173,174,175,176,177] based on magnetization transfer phenomena.

MPF parameter has been demonstrated to have a strong correlation with myelin content assessed by histology in the brain of healthy rats [178], cuprizone-induced demyelination [179,180], and ischemic stroke [139,142]. Recent studies have shown that MPF can be considered as one of the integral biomarkers of neural tissue repair, based on the correlation of myelination with the functionality of new axons [138]. More importantly, the MPF mapping method was effective in clinical trials for the diagnostics of multiple sclerosis [181,182], trauma [183], schizophrenia [184], pre- and postnatal disorders of brain development [185,186,187]. Unlike diffusion tensor imaging (DTI) parameters, MPF is insensitive to anisotropy and spatial organization of brain tissue.

In summary, demyelination appears to be the possible pathological mechanism responsible for cognitive and neurological impairments after COVID-19. Future research using quantitative MRI assessment of myelin in combination with neurological and psychological studies will help to better understand the mechanisms of post-COVID neurological and mental disorders associated with demyelination.

## 8. Conclusions

Despite extensive research and clinical data related to COVID-19, studies of demyelination in post-COVID neurological complications and cognitive impairment are limited. Theoretical and experimental studies convincingly indicate that demyelination may underlie many neurological, cognitive, and mental impairments of COVID-19 complications. Probably, the reason for this apparent contradiction lies in the imperfection of the conventional methods used to assess demyelination in the clinic. For a more accurate assessment, it is necessary to use novel, more accurate MRI methods to quantify demyelination, which can detect not only large lesions, but also subtle quantitative changes invisible to the eye. Quantitative neuroimaging might bring important insights in finding an association between myelin loss and long-lasting neurological symptoms.

## Figures and Tables

**Table 1:** Neurological manifestations of post-COVID.

Study Parameters	Huang et al. [39]	Zhang et al. [38]	Graham et al. [21]	Pilotto et al. [42]	Woo et al. [27]
Time from onset, months	6.2 (5.8–6.6)	12.0 (11.9–12.4)	5.3 (3.4–6.4)	6	2.8 (0.7–3.5)
Sample size	1733	2433	100	165	18
Male/female (%)	52/48	49.5/50.5	70/30	69.7/30.3	56/44
Age, years	57.0 (47.0–65.0)	60.0 (49.0–68.0)	43.2 (11.3)	64.8 ± 12.6	42.2 (14.3)
Mild/severe COVID-19 (%)	25/75	72.1/27.9	100/0	34.5/65.5 **	100/0
**Neurological symptoms**					
“Brain fog”	N/R	N/R	81%	N/R	N/R
Headache	2%	2.3%	68%	9.7%	N/R
Myalgia	2%	7.9%	55%	29.6%	N/R
Fatigue	63%	27.7%	85%	34%	16.7%
Anosmia/hyposmia	11%	1.3%	55%	18%	N/R
Dysgeusia/hypogeusia	7%	1.4%	59%	18%	N/R
**Mental disorders**					
Insomnia	26%	N/R	33%	30.8%	N/R
Depression/Anxiety	23%	10.4%	47%	26.7%	11.1% *
Memory deficit	N/R	N/R	32%	31%	44.4%
Attention deficit	N/R	N/R	27%	31%	50%

* Severe mood swing. ** Mild/moderate + severe COVID-19. N/R—not reported.

## Data Availability

Not applicable.
